# The role of exosomes in hepatitis, liver cirrhosis and hepatocellular carcinoma

**DOI:** 10.1111/jcmm.12950

**Published:** 2017-02-22

**Authors:** Jiliang Shen, Chiung‐Kuei Huang, Hong Yu, Bo Shen, Yaping Zhang, Yuelong Liang, Zheyong Li, Xu Feng, Jie Zhao, Lian Duan, Xiujun Cai

**Affiliations:** ^1^ Department of General SurgerySir Run‐Run Shaw HospitalZhejiang UniversityHangzhouChina; ^2^ Department of MedicineRhode Island Hospital and The Warren Alpert Medical School of Brown UniversityProvidenceRIUSA; ^3^ Department of AnesthesiologySir Run‐Run Shaw HospitalZhejiang UniversityHangzhouChina

**Keywords:** exosome, HBV, HCV, liver cirrhosis, hepatocellular carcinoma

## Abstract

Exosomes are small vesicles that were initially thought to be a mechanism for discarding unneeded membrane proteins from reticulocytes. Their mediation of intercellular communication appears to be associated with several biological functions. Current studies have shown that most mammalian cells undergo the process of exosome formation and utilize exosome‐mediated cell communication. Exosomes contain various microRNAs, mRNAs and proteins. They have been reported to mediate multiple functions, such as antigen presentation, immune escape and tumour progression. This concise review highlights the findings regarding the roles of exosomes in liver diseases, particularly hepatitis B, hepatitis C, liver cirrhosis and hepatocellular carcinoma. However, further elucidation of the contributions of exosomes to intercellular information transmission is needed. The potential medical applications of exosomes in liver diseases seem practical and will depend on the ingenuity of future investigators and their insights into exosome‐mediated biological processes.

## Introduction

Hepatitis B virus (HBV) and hepatitis C virus (HCV) are two types of viruses that infect the liver and replicate in hepatocytes 1. Approximately 2 billion people are infected with HBV, and nearly 170 million people are chronically infected with HCV worldwide 2, 3. Some of them develop progressive chronic liver diseases (CLD), including hepatitis, fibrosis, cirrhosis and even hepatocellular carcinoma (HCC) 4. Although there is a highly efficacious vaccine for HBV, many issues still exist, including vaccine cost, vaccine non‐compliance, vaccine non‐responsiveness and vaccine escape mutations 5. Antiviral nucleoside analogues against HBV were developed to resolve these issues, but drug resistance remains a problem. Sofosbuvir, a nucleotide analogue of the HCV NS5B polymerase inhibitor of all HCV genotypes, is still in a phase 3 clinical study 6. HBV and HCV remain concerns for humans.

Liver cirrhosis is the 14th most common cause of death worldwide and is an increasing cause of morbidity and mortality in developed countries 7. HBV and HCV infection, alcohol abuse, and non‐alcoholic liver diseases are the main causes of liver cirrhosis in developed countries. However, HBV infection is the most common cause of liver cirrhosis in sub‐Saharan Africa and most parts of Asia 8. Liver cirrhosis is a dynamic process, and it may eventually cause decompensation and end‐stage disease requiring liver transplantation.

Hepatocellular carcinoma is the most common primary liver cancer. Approximately, 80% of HCC cases are associated with chronic HBV or HCV infection and liver cirrhosis 9. HBV and HCV infections are considered major HCC risk factors worldwide. In addition, obesity‐related type 2 diabetes mellitus, alcohol and tobacco are risk factors for HCC 10, 11, 12. According to recent studies, more than 500,000 deaths are caused by HCC annually. Meanwhile, the incidence of HCC is increasing, making HCC an important public health burden 9, 13, 14.

## Exosomes in liver disease

Exosomes were first identified in the intracellular production of small vesicles containing specific plasma membrane proteins in maturing mammalian reticulocytes 15. Before being released into the extracellular milieu, exosomes were identified intracellularly in multivesicular bodies (MVBs) 16, 17, 18. They were secreted with a diameter of 40–100 nm and a density in sucrose gradients of 1.13–1.19 g/ml, were found to have an endocytic origin and were enriched with tetraspanin molecules 19, 20, 21, 22. Exosomes were initially considered a mechanism for discarding membrane proteins, such as transferrin receptors, in mature red blood cells 15. Later, several studies revealed their role in intercellular communication without the need for direct cell–cell contact. Exosomes have the capacity to act as antigen‐presenting vesicles and stimulate antitumoural immune responses 23. Different types of viruses, such as human immunodeficiency virus, HBV and HCV, utilize exosomes to transfer signalling‐competent proteins and functional microRNAs to uninfected cells 24, 25, 26, 27, 28, 29. In addition, the literature increasingly indicates that exosomes may be secreted by tumour cells to promote tumour progression or suppress immune responses to tumours 30, 31, 32, 33. Similar to HBV and HCV, exosomes also play a role in liver cirrhosis and hepatocellular cancer. This review will mainly focus on the role of exosomes in liver diseases, including viral hepatitis, liver cirrhosis and hepatocellular carcinoma.

### The role of exosomes in HBV

HBV is a member of the Hepadnaviridae family that exclusively infects hepatocytes 34. Approximately 2 billion people are infected with HBV, and IFN‐α has proven to be an effective treatment for chronic infection 35. Li's study revealed that IFN‐α‐induced antiviral responses could be transmitted from liver non‐parenchymal cells (LNPCs) to HBV‐infected hepatocytes *via* exosomes and thus restore the antiviral state in hepatocytes. The antiviral response induced by IFN‐α could be transmitted from LNPCs to HBV‐infected hepatocytes *via* exosomes containing antiviral molecules. Exosomes mediate and enhance the anti‐HBV treatment effects of IFN‐α 36.

Recent studies highlighted the importance of exosomes in cell‐to‐cell communication 37, 38, and some specific exosomes were detected as biomarkers for different liver diseases 39. Marked and specific changes in exosome protein contents were also detected by comparing exosomes secreted by HBX‐infected Huh7 cells with those secreted by a control group 40. HCC‐related proteins, such as VCP, were detected in the serum exosomes of HBV‐infected patients. Detecting and understanding the changes associated with HBV‐specific exosomes may provide a new perspective regarding the diagnosis of HBV or HBV‐related HCC.

### The role of exosomes in HCV

**Figure 1:**
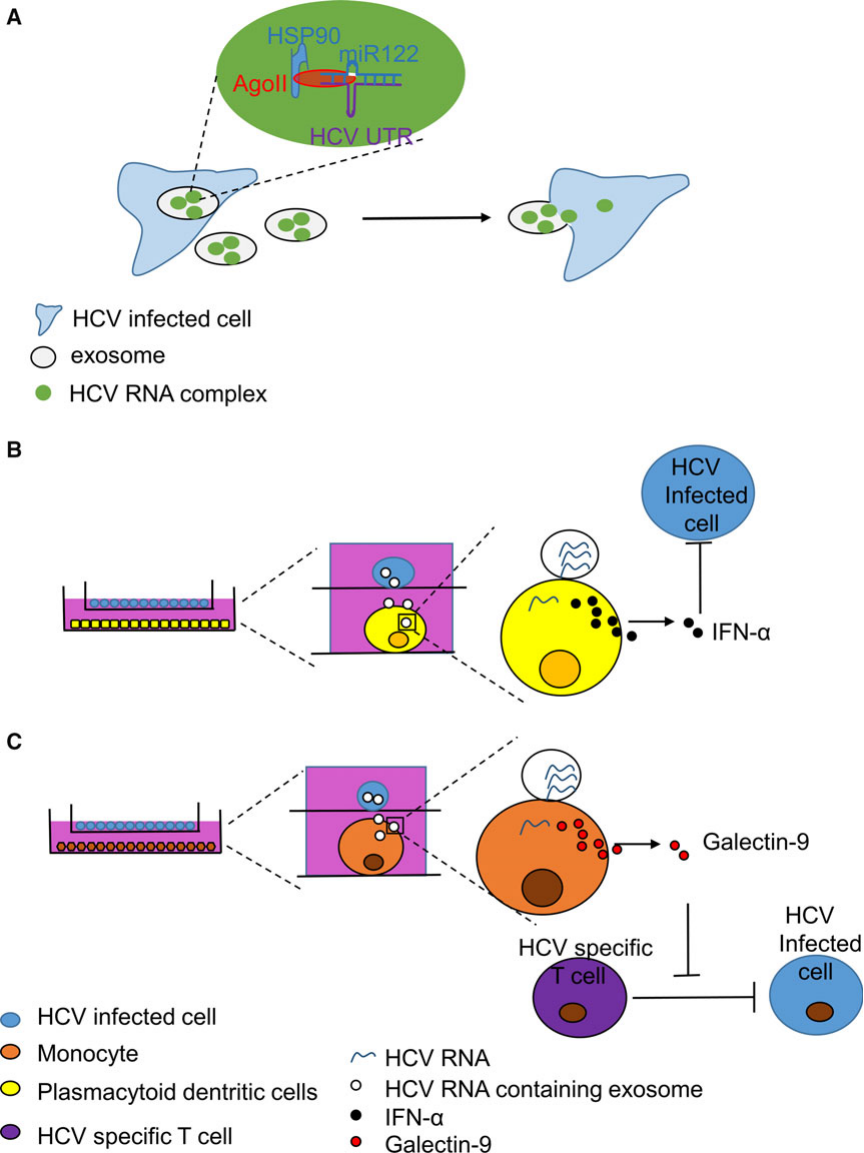
Multiple roles of exosomes in HCV infection. (**A**) exosomes mediate HCV infection *via* transmitting the HCV‐RNA complex to neighbouring cells (**B**) HCV‐RNA could be presented to PDCs *via* exosomes and activate the initial immune response (**C**) HCV‐RNA could be presented to T cells *via* exosomes and inhibit the special immune response.

Exosomes mediate cell–cell communication *via* the transfer of proteins, mRNAs and microRNAs 41. A previous study in HCV‐infected patients revealed the presence of HCV viral RNA in exosomes 28. Ramakrishnaiah's study 42 demonstrated an exosomal route of HCV transmission between hepatocytes. Further study indicated that infections involving HCV exosomes showed a higher level of HCV transmission to hepatocytes than the same multiplicity of infection (MOI) of free HCV particles 43. Some studies have suggested that exosomal HCV RNA is transmitted as a complex consisting of Ago2, HSP90 and miR‐122 and that this protein complex was proven to enhance HCV RNA stability and viral replication 43, 44, 45, 46 (Fig. 1A) . HCV exosomes are a clever strategy that may be used by the virus to ensure effective replication and may explain HCV treatment resistance with interferon. These findings may also provide new drugs that target HCV RNA‐related exosomes.

In addition to enhancing HCV transmission to hepatocytes, HCV‐related exosomes are also involved in the innate immune response and immune escape 47, 48. Plasmacytoid dendritic cells (pDCs) are a major IFN‐α‐producing cell type and have the ability to rapidly secrete type 1 IFN after being activated 49. The conventional mechanisms of pDC activation involve cell–cell contact and TLR7‐dependent interactions with the intracellular HCV RNA of co‐cultured infected cells 50, 51, 52. HCV‐infected cells could deliver their HCV RNA cargo to neighbouring pDCs by packaging viral RNA within exosomes 47 (Fig. 1B). However, the exosome transfer of HCV to pDCs occurred only when cells were in close proximity, which needs to be further studied. This new mechanism has broadened our understanding of the host–virus relationship and provided us with hints to better control HCV infections.

HCV‐related exosomes are also involved in immune escape 48. Gal‐9, a ligand of Tim‐3, could inhibit T cells, leading to immune dysfunction 53, 54, 55. Significantly higher levels of gal‐9 were detected in cultured monocytes from HCV patients than those from normal donors. The production of gal‐9 was associated with T‐cell inhibition in HCV infection. Further study indicated that close proximity and exosome release from HCV‐infected cells promoted gal‐9 secretion from monocytes (Fig. 1C). The adaptive immune inhibition associated with HCV infection that leads to viral persistence and targets specific HCV exosomes may represent a therapeutic target that enhances HCV treatment effects.

### The role of exosomes in liver cirrhosis

**Figure 2:**
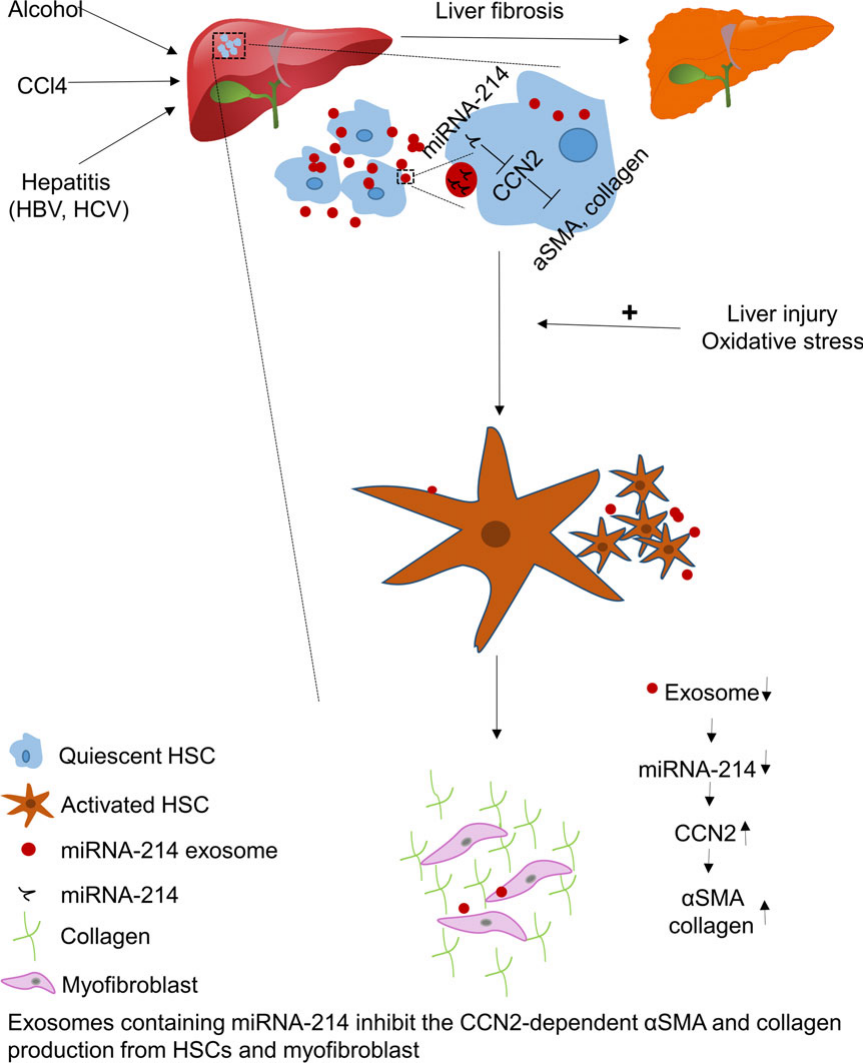
Quiescent HSCs secrete exosomes enriched in miRNA‐214 normally activated HSCs decreased the miRNA‐214 exosomes secretion and induce the enhancement of downstream pathway, which leads to liver fibrosis.

Recently, several studies have reported on the function of exosomes in the process of liver fibrosis 56, 57, 58, 59. Connective tissue growth factor 2 (CCN2), which is overexpressed in fibrotic liver, could directly promote fibrogenesis in hepatic stellate cells (HSCs) 60. The expression of CCN2 was also increased in activated HSCs compared with quiescent HSCs 61, 62. MiR‐214, which targets the CCN2 3′‐UTR directly, regulated the expression of CCN2 and was transferred from HSCs to neighbouring cells by exosomes 56. Normally, exosomal miR‐214 has a limited impact on the fibrotic response in hepatic cells. However, miR‐214 was decreased during chronic liver injury; therefore, CCN2 up‐regulation may promote liver fibrosis **(**Fig. 2). MiR‐214, a fibrosis‐related miRNA, may have potential use as a noninvasive or minimally invasive biomarker of fibrosis progression. In addition, isolated exosomal miR‐214 or mimicry may have a therapeutic effect on liver fibrosis, but the mechanism underlying exosomal miR‐214 down‐regulation in chronic liver disease remains unclear. Further related studies will be needed to verify its efficacy.

In another study 57, exosomal Twist1, which drives miR‐214 expression and facilitates CCN2 suppression in recipient cells, was reported to be suppressed during HSC activation. Suppression of exosomal Twist1 played a positive role in CCN2‐dependent fibrogenesis. CCN2 could also be packaged into secreted exosomes by activated HSCs and transferred to other quiescent or activated HSCs 58. The exchange of exosomal CCN2 may result in amplification of fibrogenic signalling in response to chronic liver injury. Targeting exosomal CCN2 may alleviate fibrosis progression.

During liver fibrosis, different exosomes are secreted to mediate fibrogenic signalling. Some exosomes may be specific to this process and can be used as biomarkers of liver fibrosis in the future. Some exosomes may be potential therapeutic targets. However, related studies are still rare, and further elucidation of the relationship between exosomes and liver fibrosis is needed.

### The role of exosomes in hepatocellular carcinoma

**Figure 3:**
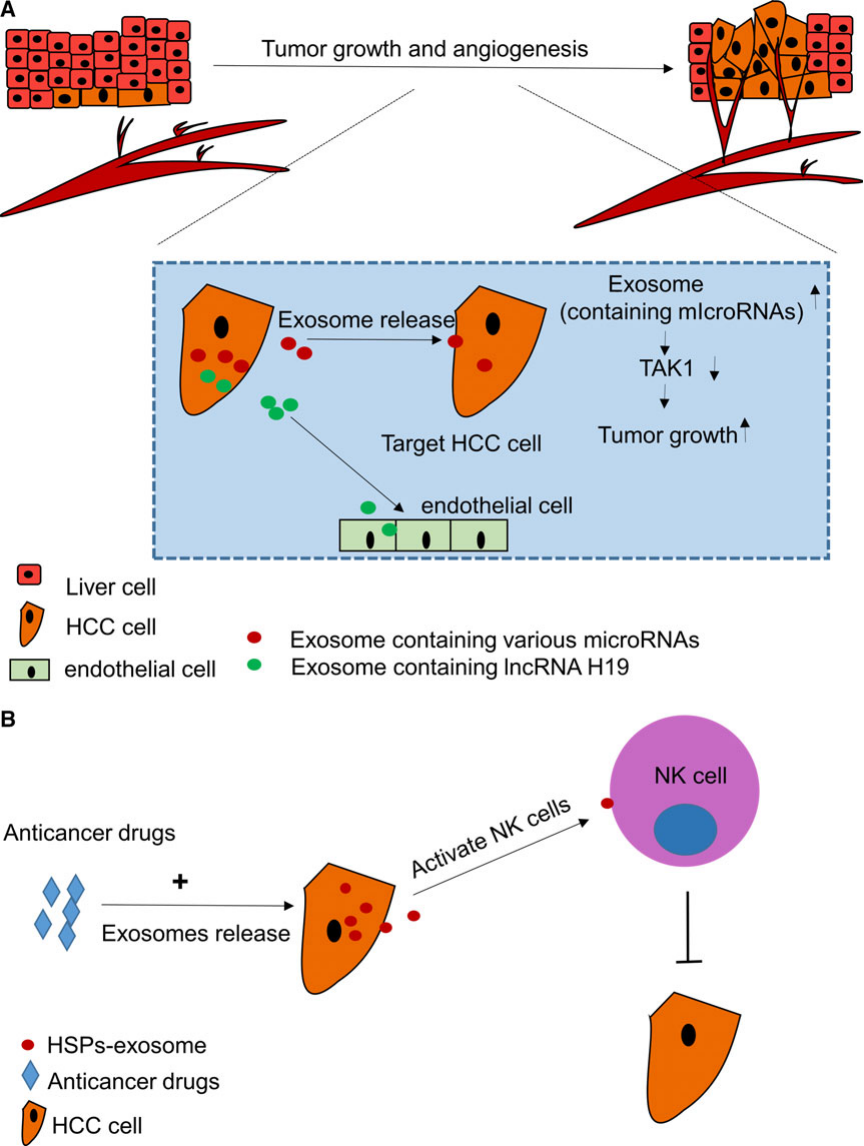
(**A**) HCC cells secret various miRNA exosomes targeting the TAK1 to promote HCC cells proliferation and secret lncRNA exosomes to promote tumour angiogenesis. (**B**) exosomes transmit HSPs to NK cell to enhance its anti‐tumour bioactivity. HSP, Heat shock protein; NK, natural killer.

Hepatocellular carcinoma is the fifth most common cancer in the world and the third most common cause of cancer‐related death 13. Genetic alterations in oncogenes and tumour suppressor genes have been extensively studied. However, the cellular microenvironment, including exosomal communications, remains less well understood. Previous studies have reported that exosomes are involved in HCC progression 63, 64. Takayuki et al. 63 reported that HCC secreted exosomes containing various miRNAs. In terms of exosome‐mediated intercellular signalling, these miRNAs mainly modulated TAK1 expression and signalling to promote development and progression of HCC. In addition, CD90+ HCC cells also promote angiogenesis in tumours *via* secreted lncRNA H19 exosome‐mediated effects on endothelial cells 64 (Fig. 3A). Targeting these exosomes may better inhibit HCC progression.

Exosomes also play a role in mediating the effects of anti‐tumour treatments. Using the exosomes secreted by adipose‐derived mesenchymal stem cells (ADMSCs) to treat HCC in rats demonstrated significant suppression of HCC development. The anti‐tumour response was mainly mediated by natural killer (NK) cells and could be enhanced by ADMSC‐derived exosomes 65.

Heat shock proteins (HSPs) are a family of highly conserved proteins 66. Anti‐tumour immunity was enhanced after the host's cellular immune system recognized tumour‐derived exosomal HSPs 67. After treatments with anti‐cancer medicines, HCC cells delivered HSP‐enriched exosomes to NK cells to enhance their anti‐tumour bioactivity 68 (Fig. 3B). HSPs expressing tumour exosomes may represent a promising alternative approach to the treatment of HCC.

## Summary and future prospects

More and more studies have revealed the roles of exosomes in liver diseases. The functions of exosomes mainly depend on the cells that receive exosome signals and contents 22, 69, 70, 71. Current studies have presented the multiple functions of exosomes in liver diseases. In HBV treatment, exosomes transmit the antiviral response from LNPCs to HBV‐infected cells 40. HCV‐infected cells secrete HCV RNA‐exosomes, whose functions lead to infection (to hepatocytes) or initial immune activation (to pDCs) depending on where they are transmitted 46, 47. Exosomes also play a role in the process of liver fibrosis by mediating communication between HSCs and hepatocytes. Decreased exosomal miR‐214 is mostly detected at the initial stage of liver fibrosis and promotes the fibrotic process. However, the mechanism underlying how these exosomes are down‐regulated needs to be further studied in the future. HCC cells could secrete exosomes to deliver miRNA or lncRNA to surrounding cells, including other HCC cells or endothelial cells. Cell–cell communication mediated by these exosomes may promote tumour growth and angiogenesis 63, 64. However, in anti‐tumour treatment, HCC cells also secrete exosomes with enriched HSPs, which promote NK cell anti‐tumour bioactivity 68.

In summary, one category of exosomes may be related to the development of liver diseases and may be valuable in diagnosis. Another category of exosomes plays a role in promoting hepatitis, cirrhosis and HCC. Targeting different types of exosomes may help clinicians to better control liver diseases. However, further studies will be needed to gain full knowledge of exosome formation and function to better utilize their capabilities.

## Conflict of interest

All authors confirmed that there is no conflicts of interest.
